# Book and Pamphlet Notices

**Published:** 1858-05

**Authors:** 


### BOOK AND PAMPHLET NOTICES.

On the Use of Iron. By Isaac Casselberry, M. D., Evansville, Ind. Extracted from the *American Journal of the Medical Sciences* for April, 1858.

Dr. Casselberry is a hard worker in the profession. He has given great attention for many years to the pathology and therapeutics of fever, and recently produced two excellent papers, which have been extensively noticed in a very favorable manner by our cotemporary journals. They were on the Use of Quinine in Fever, and the Use of Water in the same disease. This last pamphlet of twelve pages contains much valuable information on the use of iron in fever and its sequela, and should be read extensively by the juniors of our profession in the west. We know no better way, however, to impress our readers with the merit of this production, than to present them with the following disconnected extracts. As to their discriminating judiciousness and practical utility, there will be but one opinion with the experienced.

After the employment of appropriate remedial agents, and the removal of the more manifest symptoms of fever, there often remains an impoverished condition of the blood, which is a fertile source of local dependant forms of disease, especially when, during the progress of fever, a chill from time to time occurs. This condition of the blood is *sensitively* evinced by lesion of nutrition, loss of nervous energy, want of appetite, muscular debility, and more or less perversion of secretion; and *it is caused by deficient developmental intensity of the molecular combinations of the elements of the blood.* * * * * * *

From the physiological fact that the globules do not contribute to the nutrition of the tissues until they have attained maturity; that they will not mature normally without certain increments of iron; that when these are normally present they greatly promote the developmental intensity and activity of the blood-cells; that they increase the capacity of these cells for the absorption of the oxygen of the atmosphere and for the secretion of carbonic acid gas from the blood, by which the globules assume a bright-red color; that the blood in this manner oxidized is conveyed and introduced into the capillaries, in which its elements are transformed, matured, and appropriated to the nutrition of the tissues, evolves animal heat, and absorbs the effete constituents of the transformed tissues; and that the pathological states which are produced are those which are dependant on imperfect molecular development of the globules, and the consequent deficient oxidation and depuration of the blood; we can appreciate the causes of the perverted condition of the different forms of the automatic nervous force, the abnormal forms this force assumes in the molecular combination of the elements of the blood, when all development is deficient, and the best means to effect its tranquility and early restoration.

Iron is the most efficient agent to promote the normal restoration of these lesions, because it supplies the element required to promote the growth and maturity of the protein globules. The effects of few medicinal substances are more immediate or more remarkable than that which results in disease from deficient cell development from the exhibition of iron. * * * * *

Several of the compounds of iron are often given in larger doses than is necessary when the design is to promote the absorption of the iron into the blood. This observation applies especially to the carbonate, sulphate, and muriated tincture. The regeneration of the globules, when much diminished in quantity and altered in quality, must require considerable time.

That the efficacy of a ferruginous compound is not in proportion to the quantity of iron it contains is shown by the fact that many mineral waters are very powerful, though they contain less than a grain in the pint. This fact clearly evinces the necessity of the greatest care in the selection of the compound we are about to employ; because the efficacy of iron often depends on the compound used and its mode of administration. Deficient cell-growth, which occasions the necessity for the employment of iron, causes a vast multiplicity of symptoms, which are produced by the functional disturbance of the visceral glands. The compound of iron should, as nearly as possible, be adapted to the particular state of the digestive organs, that it may be readily absorbed and elaborated with the nutritive elements of the blood; for this is the only mode by which iron can promote the growth and maturity of the blood. Sir James Murray first recommended the administration of iron in the following mode: Dissolve one drachm of the bicarbonate of soda in four ounces of water; then add to this one drachm of the muriated tincture of iron. The draught should be taken during effervescence. It should be repeated three or four times a day. Although the quantity of iron is small, yet it is in a state of subdivision so minute as to favour greatly the absorption of each increment. The double decomposition which takes place forms, as one of its products, muriate of soda. This saline is most congenial to the development of the globules.

During the protracted continuance of fever, diarrhœa, dysentery, or any other form of disease, during the autumn or winter in the Southwest, the use of iron according to this suggestion of Sir James Murray, is often attended with the greatest efficacy, especially when the fever, or other form of disease, assumes what is usually termed a typhoid fever. The iron should be administered every three or four hours, alternated with other appropriate remedies. The minute quantities of iron and muriate of soda thus presented to the digestive and absorbant glands, which have been so long deranged and weakened, stimulate and promote the growth and maturity of their cells, and thereby favor the digestion and accelerate the absorption of any nutritive or medicinal substance. This will be clearly evinced by the increased secretion, which will take place in a day or two from the beginning of the use of the iron. The biliary, urinary, and cutaneous secretions will be greatly augmented; the tongue will become more moist, the thirst less urgent, and the sleep more tranquil.

An Essay on Prolapse of the Funis, with a New Method of Treatment. By J. Gaillard Taylor, M.D., Physician to the Demilt Dispensary, New York City. Read, February 3d, 1858, before the New York Academy of Medicine.

This paper is intended to illustrate the importance of posture in the treatment of cases of prolapsed funis. It will be recollected that the axis of the superior pelvic strait and the uterine cavity is represented by a line drawn from a point just above the umbilicus to the lower end of the sacrum. When, therefore, a parturient patient is supine, the umbilicus pointing from the most prominent part of the abdominal tumor, the axis of the uterine cavity will be represented by a line drawn at an angle of forty-five degrees above the horizon, and the sacrum and posterior wall of the uterus form an inclined plane, lubricated to vexatious slipperyness, down which the cord persists in sliding, despite repeated efforts to return it. Dr. Taylor, by causing the patient to assume the prone posture, with the knees drawn under her, suspends the umbilicus at the most dependent point of the abdominal tumor, and thus makes an inclined plane of the pubis and anterior wall of the uterus, *conveniently* slippery to allow the cord to slip toward the fundus uteri. It will be seen that by this management we counteract the most efficient cause of prolapse, and promote, by the power of gravity, its tendency toward the fundus instead of the os uteri, and cause it to fall *up* instead of *down*, so far as the *via-parturientis* is concerned.

Dr. Taylor suggests the following rules for our government in what he calls very aptly the postural treatment of prolapsed funis:

"1st. If the cord be detected in the unruptured bag, the woman should be placed in position before the water escape, and that no efforts at return of the prolapsed part be made by the hand. The position alone will, I believe, cause its return to the uterus, and if it does not, we may do so manually as soon as the waters escape."

"2d. That if the pelvis be so fully occupied by the presenting part as to preclude return of the cord by the hand, a gum-elastic catheter and tape to be used as a porte-cordon."

"3d. No manipulation should be commenced until the woman be placed in position."

"4th. In returning the cord, the whole hand should be introduced into the vagina. This is essential to success; the finger alone will fail."

This is an ingenious and we think promising method of remedying a perplexing and dangerous class of labors.

The pamphlet is illustrated by two drawings, which demonstrate very conclusively the philosophy of the premises upon which the valuable suggestions of Dr. Taylor are based. One of the figures is the median section of a patient lying on her back; and the other the same turned on the face, with the hips elevated by placing the knees perpendicularly under them. We are thus explicit, because we desire to enable our readers to avail themselves of this method of treating such cases.

Lectures on the Sulphate of Quinia, delivered in the regular course of the Med. Depart. of the University of Michigan. By A. B. Palmer, M.D., Prof. of Materia Medica and Diseases of Women and Children. Published by the Class.

These five lectures were singled out by the class from Prof. Palmer's excellent course of instruction for last winter, as deserving of special attention from the Western student, and published. We think they were deserving of that compliment, being well written, full of useful information in regard to the therapeutical application of that drug, and fully up to the times in the many authorities upon the subject.

Dysentery, its Pathology and Treatment: Clinical Lectures at Jackson Street Hospital. By Robert Campbell, A.M., M.D., Demonstrator of Anatomy in the Medical College of Georgia, and Lecturer on Clinical Medicine at Jackson Street Hospital.

This paper of Dr. Campbell's is written with considerable care, and evinces much experience and thought, and will reward anybody who may take the time to read it. By the way, why is it that so many remedies of very different character are praised as being extraordinarily successful in dysentery? Because all cases and epidemics are not alike, and are not amenable to the same treatment. That only half explains the difficulty. Two physicians in the same vicinity, at the same time, during the same epidemic, and as far as can be judged in the same class of cases, will pursue a very different, nay, to all appearances, entirely opposite course of management, and yet succeed probably equally well. Can these facts be accounted for on the principle of one powerful impression calling off the nerve force and vascular momentum from the parts affected, and thus weaken the morbid action? And thus allow in reality of more than one right way to perform a cure? We think so. We believe this therapeutic means of counteracting one impression by inducing a series—one after another—of different impressions, by medicines, by mental action, change of habits, a revolution in the state of things, out of which normal action may be instituted, through the recuperative efforts always made by the system under all possible circumstances, is too often lost sight of when it might be made available. Salts, quinia, tannin, opium, acet. lead, cold water, ice, in fact, the thousand and one remedies that might be named that have cured this disease, cannot produce the same physiological impression on the great vascular, nervous, and secerning systems, and yet they may all cure. The secret of their operation is: they all do produce an impression, not on the part diseased directly, not on the blood in a manner calculated to heal the bowels, but on the nervous, secretory, and vascular systems, all in such a way as to embarass the morbid action, by inducing a condition in them all different from what existed in the beginning of the disease. Young physicians are often puzzled to know why Dr. A. does not treat the same disease just like Dr. B. The reason is that he has learned to cure disease by different methods, probably just as well, but certainly not in the same way. This comprehensive view of the *methodus medendi* intelligently carried out will relieve us from the disagreeable routinism of prescribing this remedy for that disease, and expecting invariable results from the same thing under what appears the same circumstances. We may select different modes of attack by sapping and mining, scaling, or by direct fire at the object. We will seldom, however, be in possession of the remedy that after entering the mouth and stomach proceeds directly to the part affected, but must be conducted around it until it is undermined and overturned.

